# National Dental Association, Niagara Falls, August 1–4, 1899

**Published:** 1899-11

**Authors:** J. M. Walker


### PROCEEDINGS.

### National Dental Association, Niagara Falls, August 1-4, 1899.

Reported for the DENTAL REGISTER by Mrs. J. M. WALKER.

### PORCELAIN ENAMEL INLAYS.

BY N. S. JENKINS, D.D.S., DRESDEN, GERMANY.

The process and material of the Jenkins porcelain enamel is the result of seven years' study and experiment, scientific experiment and practical application going hand in hand. The successful use of this finally completed method, for over two years, justifies the claim that the problem of making, with mathematical accuracy and scientific certainty, absolutely perfect fillings in diseased teeth has been solved. A perfect filling, as thus understood, is one that fills the cavity so exactly as to exclude moisture; of a substance which will not disintegrate nor change its original form, either through chemical action or mechanical force; with a surface so smooth that it can be easily kept clean; a poor conductor of caloric; retaining the color and shape of the tooth; applicable to the most desperate cases and susceptible of being used without too great strain upon timid children and delicate patients, and its working must not make too great drafts upon the strength and nerves of the operator. It must be possible for any good dentist to use it with the certainty of obtaining infallible results. All these qualities are found in the porcelain enamel. A substance which can be molded in a gold matrix is necessary, in order to permit its use by any competent dentist, in cavities in any part of the mouth, as an ordinary, well-regulated proceeding in daily practice. Only the most exceptionally gifted and patient man can obtain such results with platinum, because of its intractibility. To keep the gold foil impression exact during fusing, a paste of powdered asbestos and water is found best adapted to hold it in place, evaporating the moisture gently by drying out. The heat of gas is sufficient for fusing this body. It fuses at a temperature of 800° to 900° C., the melting point of gold being about 1075° C. The fit of the inlay is so exact that only a slight film of cement is required, but it is important to groove the inlay with a small diamond disk, and to form small undercuts in the cavity before setting the inlay. It is well to saturate the cavity with carbolic acid before setting the inlay, to render the pulp less sensitive to the irritating action of the phosphoric acid. A cement with very fine powder is to be preferred. The powder composing the inlay should be mixed with absolute alcohol, which evaporates with less disturbance of the particles than water, and also because it carries no deleterious substances in solution. The porcelain enamel adheres fairly well to platinum, and when roots are to be banded for crowns the visible portion of the band can be covered with the enamel to great advantage.

DISCUSSION.

In the discussion of Dr. Jenkins' paper, Dr. John I. Hart said that the interest manifested in this high class of work is a refutation of the idea that dental science is drifting toward commercialism; that it is lacking in artistic refinement. As a matter of personal choice Dr. Hart prefers a high fusing body, in a platinum matrix, which, if properly annealed, does not offer the difficulties portrayed in the paper. The advantage of the platinum matrix is that it can be tried in the cavity and more body added if necessary. For making a platinum matrix, in deep cavities, Dr. Hart's method is to take an impression of the tooth and cavity in modeling compound and obtain a replica of the tooth and cavity in oxyphosphate of zinc, which will stand the burnishing of the platinum into the cavity and over the margins. By filling this matrix two-thirds full and when cool carrying it to the cavity in the tooth, it can be pressed home, thus securing a perfect adaptation.

Dr. Darby said that he has had ample opportunity of observing Dr. Jenkins' artistic methods, which, in accuracy of fit and perfection of color, perfectly fulfill the needs of the case. By this method a perfect inlay can be made, and it is one of the greatest of recent improvements in dental practice. It is not a method for the careless man, but any man who is competent to practice dentistry can, with painstaking efforts, obtain results which will be eminently satisfactory to both himself and his patients.

Dr. C. N. Johnson, while admiring Dr. Jenkins' clearly defined, easily understood description of his method, desired to utter a word of caution, as it must not be supposed that this method is one to be readily picked up by the average practitioner. There is nothing in operative dentistry that requires such absolute accuracy, in the minutest details, as this work. An ordinary operator will have a gold filling completed in less time than is necessary to prepare the cavity and make the matrix for porcelain inlay, and the gold filling has the advantage of a perfect joint as there is no cement to dissolve out. It is there to stay.

Dr. Joseph Head said that as to permanency, we know that inlays have lasted, and do last; even the old glass fillings, which discolored under the action of the saliva, have lasted eight and ten years, and bid fair to last as much longer. The average life of a gold filling is not over five years, and why should we ask more from porcelain inlays? As to the difficulties to be encountered, let any man look back and recall his first gold filling, and he will not expect success for his first inlay. The only question about the Jenkins' porcelain is its durability. It is only two years old in its present perfected form, and both the method and material are *on trial* as yet; we are not prepared to pass judgment upon it. It is a low-grade fusing body, and in a gold matrix. The possibilities offered by the platinum matrix of a second burnishing seems very desirable. When platinum is properly annealed there is no difficulty in obtaining a perfect matrix. With mechanical skill and due care either method will give good results, but in the long run it would appear that a high fusing body in a platinum matrix will give the greatest satisfaction.

Dr. George Evans spoke of the great advantages offered by Dr. Jenkins' material in enameling the labial surface of all gold crowns. The gold crown, with properly strengthened cusps, offers great advantages in perfect adaptation, and when enameled in the exact shade required, as can be done with the Jenkins porcelain enamel, the result compares favorably with the English tooth.

Dr. McKellops said that for the corners of front teeth nothing can equal platinum-gold; nothing can touch it. You can't wear it out; with nothing else can you accomplish such grand results. Porcelain inlays can probably be made with greater ease and comfort, but when you want durability, platinum-gold is the thing.

Dr. Ottolengui said that it was proposed that in Dr. Jenkins' clinic duplicate cavities be prepared and fitted respectively with inlays made with the Jenkins porcelain enamel, made in a gold matrix, and with a high fusing body in a platinum matrix. Only by such a comparison could the question of relative superiority be definitely decided.

Dr. Jenkins said that the special claim he made for his method was its simplicity, without in any way questioning the fact that equally good results could be obtained by other methods, but at the expense of more time and greater labor. Restorations in porcelain enamel are the nearest approach to nature possible to dental art.

### THE RADICAL CURE OF CLEFT PALATE.

BY TRUMAN W. BROPHY, M.D., D.D.S.

While the methods originating with Dr. Brophy are not yet generally practiced, and have been severely criticised, it is by those who do not fully comprehend them, many of the most distinguished surgeons who formerly questioned them, being now their most enthusiastic advocates.

Dr. Brophy said that his first cases were undertaken with trepidation as his method was a transgression of all the long-accepted rules of surgical procedure. But the question of early operation has passed the experimental stage. The results of hundreds of operations performed at from ten days of age to three months have fully justified the practice.

The reasons for operating as early as practicable after birth are:

First. The surgical shock is less, as children react better; all mental apprehension is eliminated, alarm and dread being among the most powerful factors in producing shock; the nervous system is not well developed in the infant and they are not capable of receiving the impressions that they would later in life.

Second. Before the bones are fully calcified they may be bent or moved without full fracture, and hence the injury is really less than after more complete calcification.

Third. If the muscles are very early brought into action they develop instead of atrophy, and hence a good velum is secured, with plenty of tissue. Later in life, after the parts have shrunken through non-use, they can rarely be made to subserve the same purpose as when developed through natural employment.

It was predicted by eminent surgeons that the upper jaw being reduced in breadth, as the result of Dr. Brophy's operation, it would always remain contracted and the superior teeth erupt, if at all, within the inferior arch; but it has invariably been the case that the arch has spread, and then assumed nearly or quite their usual relations, all the tissues developing according to accepted types.

A fourth advantage is that the operation being performed before faulty habits of enunciation have been acquired, normal speech is assured. The operation upon the palate should always be made before operating upon the lip, thus allowing free access, as all the space that can be secured is required.

The soft bones of the hard palate and alveolar process of young patients are easily cut through with the knife. It may be necessary, in order to close the fissure, to divide the mucous membrane and bone through the malar process, dividing a maximum amount of bone and a minimum amount of membrane, the bone being then readily moved toward the median line and the cleft closed by approximation of the two sides. If the parts are kept antiseptically clean they will unite kindly, and the palate be restored so that its full function may be performed. The alveolar process developing with the teeth is a pronounced factor in the formation of the jaw, guiding the teeth into proper position.

In connection with the reading of this paper, Dr. Brophy had present some of the patients who had been operated upon at a very early age. One, a girl, now ten years old, who was operated upon at the age of ten days, having double hare-lip with a wide cleft of both hard and soft palate. The fissures of the lip extended into the nostrils, the intermaxillary bone and the central portion of the lip being rudimentary.

Another was a babe of three months, which had been operated upon at the age of three weeks for single hare-lip and cleft palate. This little patient is a brother of the girl first mentioned, four of six children of the parents being affected. Of the six children, the oldest was entirely free from deformity. The girl, above-mentioned, was the second born; the third had a hare-lip; the fourth a double hare-lip and cleft palate; the fifth child had no deformity; the sixth being the babe presented. The paternal grandfather was similarly afflicted, confirming the opinion that nearly all such cases are of hereditary origin.

DISCUSSION.

In the discussion of this paper, Dr. W. C. Barrett said that if dentistry had never given anything to the world but this operation for the radical cure of cleft palate, dentistry would be immortal. It is an operation that stands on its merits and claims the approbation of the world. It is the very climax of all operations in the cases to which it is adapted. By the operation the vault is necessarily very much reduced in breadth, but by some mysterious operation it expands and the deciduous teeth erupt normally and in nearly if not quite complete occlusion. It takes on the type of normality and the child speaks plainly.

Dr. M. H. Cryer said that at an appropriate time he would demonstrate why the jaw is not narrowed by this operation. The jaw is normally smaller in infancy and old age; the spreading is done by the bone which carries the teeth.

### A RESUME OF THE IMPORTANT CHANGES THAT HAVE TAKEN PLACE IN DENTISTRY DURING THE LAST THIRTY-FIVE YEARS.

BY DR. J. N. CROUSE.

In this paper Dr. Crouse reviewed briefly the methods in vogue before the introduction of the rubber-dam, the use of cohesive gold and the mallet and the invention of the dental engine. He described the Arthur system for the prevention of decay, and showed the importance of the restoration of full contour, although shallow cavities in the proximal surfaces of the six anterior teeth may often be advantageously obliterated by judicious cutting from the proximio-lingual surface. In the majority of cases decay will not occur. He briefly reviewed the work of Dr. W. D. Miller in the discovery of the real causes of dental decay; also the "New Departure" theories. The conclusions drawn by Dr. Crouse from his review of the past thirty years are:

First. That the practice of filling with non-cohesive gold, in the form of cylinders, is too valuable to be abandoned, especially at the cervical margins of large proximal cavities.

Second. That heavy gold can be packed within a cavity more uniformly and with less pressure than lighter gold formed into blocks.

Third. That when the ravages of decay are the most active and the walls very frail, gold is the most valuable material to use.

Fourth. That in very many cases gold is not so desirable, not because the teeth are too soft, but because the peridental membrane is likely to be disturbed and injured.

Fifth. That it is wise practice to protect the danger-points by extension of the cavity when decay is likely to occur beyond the filling.

Sixth. That the occluding surface is impaired by open spaces; this should be overcome by contouring.

Seventh. That the great question yet unsolved is to ascertain the cause of dental caries; what conditions cause the radical difference in different mouths, or in the same mouth at different times.

DISCUSSION.

Dr. S. B. Palmer, having been called upon to open the discussion of this paper, said that in teeth where the dentin is undeveloped and receiving support from the pulp, the conditions are changed in the presence of gold; the condition becomes abnormal; the dentin ceases to calcify in contact with a filling which is a non-conducting medium. Gold does not have this effect in a tooth of mature normal structure, but in teeth of a low grade, with highly organized dentin, calcification is checked; calcic deposits cease, and decay continues around the filling. Under proper conditions gold preserves the tooth, but not in the conditions named.

Dr. G. V. Black said that the welding property of gold should be thoroughly studied by every student in our dental schools. Chemical experiments should be made and our younger men given a better understanding of the properties of gold. There is much in the old books which is worthy of careful study, and unless we connect the present with the past, we can not judge what are the waste products of thought and what holds good. The present is but the past made more perfect.

Dr. J. Y. Crawford said that the study of the etiological factors in caries—this disease which has the elements of infection and contagion, and which will yet yield to the great law of immunity—we must settle, if possible, the etiological significance of caries, and its therapeutics, from the standpoint of the law of immunity. We know that there is such a condition as partial immunity, and if partial immunity is possible, perpetual immunity must also be a possibility.

Dr. G. E. Kells spoke of the importance of carefully preparing cavities which are to be filled with amalgam.

Dr. McKellops spoke of the value of a foundation of oxyphosphate cement, if you are going to use amalgam at all. Decay will not recur under oxyphosphate if it is properly put in.

Dr. H. B. Noble described a case in which the Arthur system was adopted with good results—a shallow cavity in a left superior lateral which was dressed down for the lingual surface in 1854. He was threatened with a suit for damages for having cut away a portion of the tooth, and Dr. Maynard and other eminent dentists of that day thought he had done wrong. The result has justified the means, for that tooth is perfect to-day, while other teeth in the same mouth, filled at that time, have been filled and refilled and are badly broken down.

Dr. W. C. Barrett said that as he grows older, and perhaps lazy, he finds that he can satisfy his patients and his own conscience with other materials than gold, and believes that he has done his full duty when he preserves the integrity of the tooth, though gold is, after all, the only thing that really satisfies the inmost longings of the soul of the honest dentist. But there are many cases where the next best thing, and that is, the plastics renewed again and again.

Dr. Crouse, in closing the discussion of the paper, said the discussion of the cements was not one of the objects he had in view in writing the paper, as that subject would come up later in connection with other papers. But he wished to emphasize his first proposition—that "cylinder filling" is almost a lost art, but one that deserves to be revived. He wished to make a special plea for the use of gold in the most difficult cases. When we have frail walls, there we want cohesive gold. There is nothing like gold to keep the profession up to the standard as skilled operators. As regards cutting away superficial decay, Dr. Crouse said that he recently removed a lateral and a central incisor in which twenty-eight years ago he filled one side and cut away the other. The filled cavities have had to be refilled but the side that was cut away has never decayed. He said—in all my experience there has not been, to my knowledge, a recurrence of decay when it was early removed by cutting away and polishing the surface. It don't come back.

